# Assessing judges' use and awareness of cognitive heuristic decision-making

**DOI:** 10.3389/fcogn.2025.1421488

**Published:** 2025-03-26

**Authors:** Amaryllis-Chryssi Malegiannaki, Athanasios Chatzopoulos, Konstantinos Tsagkaridis

**Affiliations:** Department of Psychology, School of Humanities and Social Sciences, University of Western Macedonia, Florina, Greece

**Keywords:** heuristics, cognitive bias, awareness, reflective thinking, metacognition, decision-making, judicial, assessment

## Abstract

Heuristics, characterized as concise cognitive shortcuts rooted in intuitive reasoning, are both capable of facilitating swift judgments and cognitive efficiency, but also introducing cognitive biases during decision-making. The judicial domain, renowned for its demanding decision-making processes, is an interesting field for studying heuristics. In this study, we developed a novel Judicial Heuristics Assessment Questionnaire (J-HAQ) and administered it to a sample of 52 judges (20 males, M_age_ = 45.50, SD = 8.10), with active duty in various courts across Greece. We also evaluated their analytical System 2 thinking skills using the Cognitive Reflection Test (CRT). This research pursued three objectives: (a) to explore the psychometric properties of the J-HAQ; (b) to investigate the correlation between judges' perceived use of heuristics/metacognitive awareness and their objective performance on reflective thinking; (c) to assess the correlation of self-reported usage of different heuristics and explore the influence of judges' demographics (educational level, gender, age, and years of experience) in the utilization of the reported heuristics in decision-making. Findings from a Principal Component Analysis on J-HAQ scores revealed four distinct factors (Availability, Confirmation Bias, Representativeness, and Anchoring) demonstrating sufficient reliability. We also report a significant correlation between CRT scores and reported use of the anchoring heuristic (ρ = 0.29, *p* = 0.04). Finally, we discovered two clusters defined by different awareness of the use of various heuristics, as well as significant association of educational level with this usage. Despite the limitations of a relatively small sample size, these findings reveal a dynamic for further interesting results from research in this domain.

## 1 Introduction

Research has shown that the way in which judges, a critical component of the legal system (Anderson et al., 2012; Gilovich et al., 2002), formulate judicial decisions, apply rules, or determine sentences, is influenced not only by the evidence and information presented during legal proceedings but also by underlying cognitive processes that play a significant role (Peer and Gamliel, 2013). In this frame, judges could be viewed as active information processors and judicial decision making can be considered an applied area for exploring theoretical concepts related to reasoning, judgment, and decision-making. This aspect has drawn attention from research within both cognitive and social psychology fields, where these disciplines could also offer insights to judges, enabling them to follow fairer procedures and achieve more favorable outcomes (Berthet, 2022; Zong and Guo, 2022). Consequently, there exists a dialectical relationship between applied areas of decision-making and theory, with research holding implications for legal policy (i.e., see Australian Law Reform Commission, 2021).

Dual-process models of cognition, as proposed by Kahneman (2011), suggest that humans process information and make decisions based on two systems of thought. System 1 thinking involves rapid, intuitive, or experiential decision-making with minimal cognitive effort. In contrast, System 2 thinking is characterized by analytical processing, cognitive demands, and longer duration of decision-making processes (De Neys, 2018). When using System 1, prior knowledge is typically linked to new experiences based on shared features. As a result, the individual's working memory will not fully process the new information (Evans and Stanovich, 2013). Indeed, judges sometimes rely on cognitive heuristic thinking, which is associated with System 1 thinking. In the practice of the judicial profession, the decision-making process is often continuous, leaving insufficient time to thoroughly consider all aspects before reaching a verdict. Consequently, judges sometimes resort to heuristic ways of thinking (Gravett, 2017).

Heuristics are cognitive shortcuts that operate empirically, particularly under conditions of uncertainty, by considering an individual's prior experiences and common features of the decisions at hand. These shortcuts can impair validity and increase the likelihood of cognitive errors, introducing what is known as cognitive biases (Pohl, 2017; Tversky and Kahneman, 1974). It is therefore essential, when studying heuristics, to take into account their potential limitations and the associated risks of bias. The phenomenon of cognitive bias has been extensively studied across a variety of applied settings (e.g., forensic, legal, medicine, and financial) (c.f Berthet, 2022 for a review of cognitive biases in four occupational areas). Research has demonstrated that both laypersons and experts are susceptible to the effects of cognitive bias (Dror and Rosenthal, 2008; Olaborede and Meintjes-Van der Walt, 2020; Richie and Josephson, 2018).

On the other hand, in stark contrast to the pessimistic view of Berthet (2022), Gigerenzer conceptualizes heuristics as a basic and adaptive collection of cognitive techniques that are part of the decision-maker's toolbox (Goldstein and Gigerenzer, 2002). The suggested conceptualization of fast and frugal heuristics in Gigerenzer's adaptive toolbox (Gigerenzer and Todd, 1999, Gigerenzer, 2000) implies that the use of such heuristics can be considered an advantage—a less-is-more effect (LIME) saving time and effort, when adjusted to the surrounding ecosystem (Gigerenzer, 2015). For example, use of heuristics could prove to be effective in high workload situations, where they facilitate cognitive processing and decision-making to operate efficiently and economically (De Neys, 2022). Ecological theorists propose that heuristics arise from a complex interplay involving both System 1 and System 2 processes, along with emotional influences, rather than being solely dependent on either system. Intuitive thinking, like rule-based analytical thinking, guides judgments and is not inherently free from bias. Ecological theorists point to an “adaptive toolbox” of heuristics that supports both intuitive and deliberate judgments (see Da Silva, 2023). In principle, the frequency of use of different types of heuristics could be independent from one another. If on the other hand we consider the successful use of a specific type of heuristics as a demonstration of an effective use of the adaptive toolbox, we might expect a correlation with the frequency of use of other heuristics as well. Therefore, given the existence of different types of heuristics, an intriguing question is whether the likelihood of resorting to any such cognitive shortcut (heuristic) correlates with the frequency of use of other heuristics, as well as with key demographic variables (Hjeij and Vilks, 2023). To that end, previous studies report small to medium correlations between the use of different heuristics (Toplak et al., 2017; West et al., 2008).

Regarding the influence of demographic factors on cognitive heuristic usage in decision-making, a recent review by Taylor et al. (2023) suggests that there is no variation in the frequency of use of certain classic heuristics (such as anchoring, availability, representativeness) across different age groups. In a study within the medical decision-making domain by Partsounidou et al. (2023), where physicians' awareness of four common heuristics and biases (anchoring, availability, confidence, and overconfidence bias) was assessed, age effects were observed only in cases of confidence and overconfidence biases. Older physicians (aged 60–70 years) reported a higher frequency of utilizing these heuristics compared to younger counterparts (>40 years). Another factor identified as influencing heuristic decision-making is the level of education, as determined by academic achievement (Bachelor, Master, and PhD). Individuals with higher levels of education tend to adopt a more analytical thinking style (Lucena et al., 2021).

As a conclusion, similarly to other occupational areas, within the legal domain, judges frequently rely on heuristics, leading to cognitive biases in their decision-making process, despite their legal training (Dror and Rosenthal, 2008; Olaborede and Meintjes-Van der Walt, 2020; Kumar and Mailanchi, 2023). However, despite the extensive research on juror's heuristic usage (Curley et al., 2022), there remains a lack of understanding regarding the frequency of heuristic utilization specifically by judges during courtroom proceedings, with the majority of research primarily focusing on the anchoring heuristic (Berthet, 2022; Bystranowski et al., 2021).

### 1.1 Cognitive heuristics employed in judicial decision-making

Given the plethora of heuristics that can influence decision-making, our study aimed to assess the use of some of the most commonly employed heuristics identified to impact decision-making in the judicial process (Olaborede and Meintjes-Van der Walt, 2020; Peer and Gamliel, 2013). These include:

- The *availability heuristic*, one of the most widely employed heuristics among judges and other professionals. This heuristic is defined by the likelihood of something being thought about based on its ease of retrieval from memory (Tversky and Kahneman, 1974). Essentially, the more easily something comes to mind, the more likely it is to be judged as true or frequent. However, the danger of this heuristic lies in the involvement of multiple stimuli in the retrieval process. Factors influencing availability can potentially reduce the accuracy of decision-making (Farmer and Matlin, 2019). In judicial decision-making, the availability heuristic functions as a subjective assessment of the arguments and evidence presented, based on their accessibility in memory (Olaborede and Meintjes-Van der Walt, 2020).
- *Confirmation bias*, the result of heuristic thinking, in which an individual tends to confirm his or her initial belief and reinforces it with confirmatory evidence collected in such a way that it always confirms the initial belief (De Neys, 2018). Confirmation bias can also influence judges during the hearing process when they hear and evaluate the evidence presented to them in court. Specifically, judges may be biased in favor of evidence that confirms their prior assumptions and may ignore evidence that does not correspond to them (Peer and Gamliel, 2013).
- The *anchoring heuristic*, a way of thinking around a particular fact that constitutes the “anchor” (anchoring point) and further cognitive processing is always performed focusing on that particular perception (Mussweiler et al., 2004). Judges are influenced by anchors (internal or external) when making judicial decisions (Kumar and Mailanchi, 2023; Zong and Guo, 2022). When criminal sentencing decisions involve numerical quantities, they are affected by numerical anchors, whether they are minimum sentences presented by law or sentences requested or recommended by prosecutors or lawyers (Peer and Gamliel, 2013).
- Finally, the *overconfidence heuristic*, grounded in an individual's excessive confidence in the accuracy of their decisions that stems from their self-efficacy, competence, and belief in themselves (Moore and Healy, 2008). Research indicates that judges employ this heuristic, although its impact diminishes when comparing between two options or when addressing various aspects of the range of possible responses. Furthermore, male judges exhibit higher levels of over-confidence compared to women, who tend to make decisions using a more analytical approach (Soll and Klayman, 2004).

In conclusion, judicial officers' decision-making is susceptible to cognitive errors. Such errors might lead to decisions diverging from those reached under different circumstances. This phenomenon arises from intuitive thinking, and it is quite possible that judges themselves will not always be aware of using it. Given the profound impact these decisions have on the lives of defendants, there is an imperative need for the assessment of heuristic utilization and the mitigation of cognitive biases.

### 1.2 Noise as an additional source of interpersonal variability

Noise is a well-defined concept in statistics, referring to one of many potential sources of error in a measurement, usually assigned to an unwanted source of variability, such as individual differences, different measurement instruments, unwanted interference, etc. In complex systems that require decision making, such as judicial processes, noise can introduce unwanted variability, both among judges, as well as within contexts for the same judge. In this setting, Fiedler (2007) describes noise as unwanted variability in judgments that should ideally be consistent, also pointing out the complimentary role of noise to biases (Guthrie et al., 2007).

According to Fiedler (2007), the critical difference between noise and bias is that noise introduces inconsistency and unpredictability, while bias might skew decision making in a more predictable direction. On the other hand, Gilhooly and Sleeman (2022) point out that randomness is not always the cause of noise. For example, two judges using different deterministic judging rules can result in noise with no involvement of randomness. In a sense, heuristics and biases are only one potential source of noise, but other sources, such as subjectivity, complexity and ambiguity, also exist (Faigman et al., 2014). Overall, the relationship among noise bias and/or use of heuristics can be quite complex. Even though this issue is beyond the scope of this study, it is worth considering how other types of noise can interact with the use of heuristics in judicial decision making. At a systemic level, the resulting variability caused by all these factors poses a threat to the trust in the integrity of the judicial system, and might lead to inequality and legal uncertainty.

### 1.3 Challenges in the assessment of cognitive heuristics: the case of judges

The research on cognitive heuristics and biases has produced a diverse array of measures aimed at illustrating individuals' systematic tendency to rely on intuitive System 1 thinking. Exploring assessment options to detect these deviations from rational thinking aids in identifying the susceptibility of various professionals to particular decision biases and facilitates the development of de-biasing interventions (Aczel et al., 2015). The research methodology utilized for assessing cognitive heuristics has generally followed two approaches: performance assessment through objective cognitive tasks and self-report measurement. Regarding the first approach, despite the advantage of objective assessment being a direct measurement of behavior, it has its limitations. For instance, Aczel et al. (2015) highlighted several challenges associated with objective assessment methods. These challenges include difficulty in obtaining individualized scores, as underlying cognitive properties of tasks often necessitate researchers to create composite scores (i.e., Bruine de Bruin et al., 2007; Moore and Schatz, 2017; Teovanović et al., 2015). Additionally, there are concerns regarding the extent to which incorrect responses truly reflect cognitive biases, leading to questionable construct validity of these tasks. Furthermore, the reduced ecological validity of experimental scenarios, as they lack the real-life consequences of decisions, results in low motivation among participants (Evans and Stanovich, 2013). To address these challenges in assessing cognitive heuristics, contemporary approaches employ mixed techniques, incorporating questionnaires about estimations alongside objective measures. For instance, Berthet and de Gardelle (2023) developed the Heuristics-and-Biases Inventory, an open-source repository containing over 40 measures commonly utilized in heuristics and biases research.

Specifically, concerning the assessment of heuristics and biases in the legal system, the primary focus of research has centered on biases exhibited by jurors. For instance, instruments such as the Juror Bias Scale (Kassin and Wrightsman, 1983), and its evolution, the Pretrial Juror Attitude Questionnaire (PJAQ), developed by Lecci and Myers (2008), are commonly used in this regard. However, it's important to note that these tools do not specifically target cognitive heuristics but rather focus on psychological and social biases. These biases encompass aspects such as conviction proneness, system confidence, cynicism toward the defense, racial bias, perceptions of social justice, and attributions of innate criminality. When it comes to evaluating cognitive heuristics and biases in jurors, researchers have typically employed vignettes or recordings of real or fictionalized material (see Meterko and Cooper, 2022).

To the best of our knowledge, there is currently no questionnaire specifically designed to assess the use of cognitive heuristics in judges. Instead, there are a few assessments involving again scenarios and cases that serve as objective measures (Peer and Gamliel, 2013), encompassing the various heuristics mentioned above.

### 1.4 Self-reports: new insights into assessing judges' awareness of cognitive heuristic use

To address this gap in the literature, we developed the Judicial Heuristics Assessment Questionnaire (J-HAQ). We trained two judges on the concept of heuristics and their applications in everyday life. Then, using the evidence regarding cognitive heuristics commonly employed in the judicial decision-making, which we present in the relevant earlier section of this introduction, we created items referring to relevant scenarios of heuristics used in judicial decision-making, collaboratively with the two judges. The goal was to develop a tool to evaluate the extent to which judges are aware of their use of heuristics when making decisions. This focus on awareness is crucial, as previous studies have not taken into account judges' perceptions regarding the frequency with which they employ specific examples of cognitive heuristics in their professional decision-making. Judges' self-reports collected through the J-HAQ could be viewed as metacognitive individual-oriented knowledge of the frequency with which cognitive heuristics are utilized in their judicial decision-making. They therefore include measurement of not only the frequency of use of such heuristics, but also awareness of using them.

### 1.5 Metacognition in judges

According to metacognitive theory (Flavell, 1987), an individual's awareness of their own knowledge, their awareness of their cognitive system, and its individual functions is referred to as metacognition (Akturk and Sahin, 2011; Scott and Levy, 2013). Metacognition encompasses knowledge about cognition through monitoring and control of cognition via regulation. Three distinct forms of metacognitive knowledge have been identified: knowledge oriented toward the individual, the task, and strategies (Händel et al., 2013). Individual-oriented knowledge involves understanding personal motives, emotions, and cognitive processes. Task-oriented knowledge pertains to understanding the specific characteristics, difficulty level, and factors influencing the task at hand. Lastly, strategy-oriented knowledge entails understanding effective ways of managing tasks each time (Frenkel, 2014). Since these forms of awareness reflect one's own thoughts, which are not directly observable, self-report measures are the most preferred assessments [see the review by Craig et al. (2020)].

Most research on metacognition has focused on the educational domain, with limited attention to legal decision-making. Legal decision-making may be expected to be less prone to cognitive biases than that observed in studies of the general population, as judges serve as fact-finders with a strong motivation to reach fair and accurate verdicts. This high motivation for accuracy increases their vigilance and promotes more systematic processing of information, often relying on metacognitive cues (Evans, 2008). Additionally, assessing metacognitive awareness in judges is valuable because they are held accountable for their decisions, which implicitly or explicitly reinforces a need to justify their beliefs and reasoning processes (Pantazi et al., 2020).

Earlier studies (Fiedler, 2007, 2012) have shown that judges decision-making is affected by “metacognitive myopia,” a term used by Pantazi et al. (2020) to describe the tendency to overlook systematic assessment of available information. This issue is not attributed to limited cognitive ability or a lack of motivation but is instead regarded as a metacognitive failure. It reflects a lack of awareness specifically linked to the operation of various biases (i.e., confirmation bias, truth bias, e.t.c.) within judicial contexts, underscoring the importance of designing effective interventions aimed at combating them and enhancing metacognitive awareness (Pantazi et al., 2020).

It is worth pointing out that the literature shows a positive association between metacognitive awareness and decision-making (Basu and Dixit, 2022; Wokke et al., 2017). Such research studies demonstrate that higher levels of metacognitive awareness and regulation are positively correlated with analytical and adaptive intuitive decision-making styles based on the dual system process theory.

### 1.6 The present study

The primary aim of our study was to develop and assess the psychometric properties of a new self-report instrument, the Judicial Heuristics Assessment Questionnaire (J-HAQ), designed to measure judges' awareness of specific cognitive heuristics used in their decision-making processes. Moreover, we wanted to record the reported frequency of different types of heuristics by judges. Considering the prevalence of research focusing on the anchoring heuristic as particularly influential within the judicial process [as indicated in the review by Bystranowski et al. (2021)], we anticipated that it would be more frequently reported by our sample.

A second objective was to investigate the relationship between judges' metacognitive awareness, as indicated by their self-reported frequency of using these heuristics in judicial decision-making, and their performance on an analytical thinking/decision-making task, the Cognitive Reflection Test (CRT, Frederick, 2005). Building upon prior research (Basu and Dixit, 2022; Pantazi et al., 2023; Wokke et al., 2017), we hypothesized a positive correlation between judges' self-reported frequency of usage, indicative of their metacognitive awareness, and CRT scores.

The third objective of the study was to assess the correlation of self-reported usage of different heuristics and explore the influence of judges' demographics (educational level, gender, age, and years of experience) in the utilization of the reported heuristics. As in previous studies (Toplak et al., 2017; West et al., 2008), we expected to find small to medium correlations among the reported use of different heuristics by judges. It is also quite likely that factors such as educational level and years of experience will be positively correlated to the reported use of at least some types of heuristics (Gigerenzer, 2015). We nevertheless refrained from forming specific hypotheses regarding the precise influence of these demographic characteristics, due to limited and inconclusive research findings in previous studies.

## 2 Methods

### 2.1 Participants

Our sample consisted of 52 judges (20 men) from the Greek criminal justice system. Participants were drawn from 54 out of a total of 63 First Instance Courts and 11 out of a total of 19 Appeal Courts across all Greek prefectures. On average, the judges were 45.50 years old (S.D. = 8.10, min. 31 years/max. 63 years). They had an average professional experience as judges of 12.96 years (S.D. = 8.24, min. experience = 1 year/max. experience = 32 years). Most of the participants resided in urban areas (86.5%), while a small percentage lived in rural towns or villages (13.5%). Regarding educational qualifications, 18 participants (34.6%) held a Bachelor's degree, 31 (59.6%) held a Master's degree, and 3 (5.8%) held a PhD degree.

### 2.2 Instruments

For this study, two questionnaires were administered: the Judicial Heuristics Assessment Questionnaire (J-HAQ) and the brief Judges' Demographic Questionnaire. Additionally, participants completed the Cognitive Reflection Test (CRT).

#### 2.2.1 Judicial Heuristics Assessment Questionnaire

The Judicial Heuristics Assessment Questionnaire (J-HAQ) was developed specifically for this study. Initially, 19 statements were created to capture four distinct factors representing cognitive heuristics commonly observed in the judicial context: availability, confirmation bias, anchoring, and overconfidence. Participants rated their agreement with each statement on a 7-point Likert scale, ranging from 1 (strongly disagree) to 7 (strongly agree). The construction of these statements involved close collaboration with two judges who received training from researchers on the definition and application of various heuristics in real-life situations. The judges then assisted in formulating the questions and crafting specific scenarios illustrating the use of heuristics in judicial decision-making. Following the initial development, the J-HAQ items underwent a pilot testing phase with feedback from five judges. Based on their input, revisions were made to improve clarity and relevance. We used a Principal Component Analysis to select the most appropriate items to include in the questionnaire, and we removed several questions found to have poor adaptation. This process resulted in a final version of the questionnaire containing 12 items.

#### 2.2.2 Cognitive Reflection Test

The Cognitive Reflection Test (CRT), introduced by Frederick (2005), serves as an objective measure of System 2 thinking. Initially comprising three items, it was later expanded to include items 4 and 5 by Toplak et al. (2014) to enhance the assessment of reflective analytical thinking. The Greek adaptation of the five-item CRT was conducted by Liapi (2019). Cognitive tasks within the CRT are structured to elicit an automatic, intuitive but incorrect response (System 1 thinking), requiring individuals to override this response and engage in further reflection to arrive at the correct answer. Consequently, the primary aim of the CRT is to evaluate participants' capacity to resist intuitive thinking and employ analytical reasoning.

This test includes problems in the following format: “*In a pond, there is a water lily. Every day, the surface it covers doubles in size. If it takes 48 days for the entire pond to be covered by the water lily, how many days does it take for half of the pond to be covered? ____ days”* [Correct answer = 47 days; intuitive answer = 24 days]. The evaluation of responses involves assigning 1 point for each correct answer and 0 points for each incorrect answer. In the end, the sum of the participants' scores is calculated, resulting in a range of values that the participant can obtain in the test, ranging from 0 to 5 points.

#### 2.2.3 Judges' Demographic Questionnaire

The Judges' Demographic Questionnaire was developed to collect information on participants' gender, age, educational level (Bachelor, Master, PhD degree), tenure as a judge, years of experience, residency location within a Greek prefecture (village, town, or city), and whether they had been diagnosed with any neurological or psychiatric condition, which was considered to be an exclusion criterion for the study.

### 2.3 Procedure

For this study, approval was obtained from the Research Ethics Committee of the University of Western Macedonia (protocol number: 115/2023), ensuring compliance with both international and national regulations, as outlined in the principles of the Declaration of Helsinki. Participation of judges in the research process was voluntary, without any remuneration, and with explicit instruction on their right to withdraw their participation at any stage. Initially, a special request was submitted, along with all documents certifying the research and an annex of the survey tools, to the Presiding Judge of each Court of First Instance/Appellate Court for approval of the distribution of questionnaires to the Judges of the Service. After acceptance of the request by the President, the electronic form of the questionnaires was sent to the Secretariat of the Service, which was responsible for distributing them to the Judges. This method was chosen to ensure the protection of the judges' personal data and to facilitate a smooth and confidential data collection process covering a wide geographical sampling range. Out of all the invitations sent to Courts of First Instance and Appellate Courts across the country, only two were rejected, one from a Court of First Instance and one from an Appellate Court President. The entire process of completing the questionnaires and responding to the cognitive task took no longer than 20 min to complete. Data was collected between March and August 2023.

### 2.4 Methodological design

In an effort to create a valid and reliable questionnaire for the frequency of use and awareness of heuristics in the judicial process, we ran some Principal Component Analyses (PCAs) to decide which of the 19 items will be retained in the final version of J-HAQ. Then we tested its resulting factorial structure, to assess the validity of the instrument, and assessed the internal reliability of its items using Cronbach's alpha. We then calculated the mean scores of all emerging factors for J-HAQ, as well as for the CRT test, and tested their correlations, as well as correlations with some critical demographic measures (educational level, age, and years of experience). These correlations can provide some preliminary data on the ways that critical demographic characteristics, different cognitive heuristics, as well as intuitive and reflective thinking skills are associated with one another. Moreover, we ran a power analysis, following our initial findings from these correlations, to estimate the necessary sample for follow-up studies.

Finally, we ran a 2-step Cluster Analysis in our dataset, to assess whether there are specific patterns in the use of heuristics within our sample. In principle, the frequency of use and awareness for each type of heuristic, corresponding to the factors emerging from the factor analysis of the J-HAQ, could be independent. On the other hand, it is likely that a judge using one type of heuristics in the judicial process will also be more likely to use, and will have greater awareness in the use of other relevant types of heuristics. It is therefore quite likely for separate clusters to exist. This clustering analysis is useful to reveal clusters in our sample that have a different approach with regards to the overall use of heuristics in the judicial process. Complementary to the correlation data, this evidence can shed light on some decisive factors for using heuristics in the judicial process.

## 3 results

### 3.1 Analysis J-HAQ reliability and validity indicators

Initially, the questionnaire was designed to encompass 19 items across four distinct types of heuristics (five for availability, three for anchoring, six for overconfidence, and five for confirmation bias), aiming to assess the potential utilization of heuristic thinking during judicial decision-making processes. We explored the factorial structure of J-HAQ using Principal Component Analysis (PCA). The Kaiser-Meyer-Olkin (KMO) measure was 0.77, verifying sampling adequacy. Additionally, Bartlett's test of sphericity yielded a statistically significant result (χ^2^(66) = 199.962, *p* < 0.001), indicating that the correlation matrix is significantly different from an identity matrix, thus supporting the suitability of the data for factor analysis. After applying the PCA, 7 items were removed from the questionnaire due to insufficient factor loadings, and/or cross-loadings across multiple factors. Consequently, subsequent analyses were conducted on a reduced set of 12 items (Table 1).

**Table 1:** Means (*M*), standard deviations (*S.D*.), bias and standard error (S.E.) estimates, as well as 95% confidence intervals (C.I.) on the scores of individual J-HAQ questions, based on 1,000 bootstrap samples.

**Question no**	***M***	***S.D*.**	**Bias**	**S.E**.	**BCa 95% C.I. low**	**BCa 95% C.I. high**
1	2.73	1.07	0	0.15	2.44	3.04
2	1.56	0.92	0	0.13	1.33	1.83
3	1.58	0.87	0	0.12	1.35	1.81
4	1.37	0.74	0	0.10	1.19	1.60
5	2.04	1.17	0	0.16	1.71	2.36
6	3.81	0.95	0	0.13	3.54	4.06
7	1.88	0.98	0	0.14	1.63	2.13
8	2.83	1.08	0	0.15	2.54	3.12
9	2.31	1.16	0	0.16	2.00	2.62
10	4.58	0.78	0	0.10	4.35	4.77
11	3.38	1.37	0	0.19	3.02	3.73
12	2.29	1.29	0	0.18	1.94	2.65

Specifically, PCA revealed the emergence of four factors (Table 2), collectively explaining a substantial proportion (67.13%) of the total variance. Internal consistency reliability was assessed using Cronbach's alpha for each factor. Overall, the set of items exhibited high internal consistency reliability (α = 0.82), indicating robustness in measurement. The first factor, related to the availability heuristic, consisted of three items, showing satisfactory reliability (α = 0.79); all these items were originally constructed to assess the availability heuristic. The second factor, which encompassed confirmation bias, included three items and demonstrated adequate reliability (α = 0.72). Two of these items were created to account for confirmation bias items, whereas the third item (“Sometimes I am so sure of the case that I get upset when others don't see the truth that is so obvious to me”) was constructed to account for the overconfidence heuristic. Nevertheless, this statement also includes confirming one's opinion, given that the individual is certain that what they believe is the absolute truth. Additionally, the third factor, corresponding to the representativeness heuristic, consisted of three items with acceptable reliability (α = 0.60). These items were created to account for anchoring and availability heuristics. However, they were all grouped in this single factor interpreted as representativeness heuristic, as they all involved cases with probabilistic judgments. Finally, the fourth factor included three items primarily accounting for the anchoring heuristic, with acceptable reliability (α = 0.64). These items were originally created to capture a mixture of heuristics including anchoring, availability, and confirmation bias. Nevertheless, they all included a common theme: anchoring of evidence either stemming from the case itself or anchoring to the opinion of the majority/others.

**Table 2:** Factorial structure of the J-HAQ: Eigenvalues, factor loadings, and percentages of variance explained.

**J-HAQ question items**	**Availability**	**Confirmation bias**	**Representativeness**	**Anchoring**
9. If the facts of a case remind me of the facts of an earlier case I was involved in, this fact predisposes my final decision.	0.863			
5. I tried three similar political cases last week, so it is likely that the fourth case will have a similar outcome.	0.846			
8. When deeply involved in a particular case, my recollection of its details can influence my decisions in similar cases.	0.577			
7. I typically hold onto my initial opinion regarding someone's guilt or innocence throughout the case, rarely changing my stance until the proceedings conclude.		0.898		
4. Sometimes, I adhere to my initial perspective regardless of contradictory evidence that may emerge during the proceedings.		0.709		
12. Sometimes, I feel so confident about the case that it frustrates me when others fail to recognize what seems obvious to me.		0.633		
3. Sometimes, I base judicial decisions on probability even when there isn't enough evidence.			0.723	
2. The recent statistical increase in femicides in Greece inclines me to perceive the male accused as more likely guilty when compared to a female accused, given two individuals facing the same charge.			0.685	
1. When the defendant has a history of committing a certain number of offenses in the past, he/she is more likely to be found guilty in a new case.			0.620	
10. Encountering a case similar to a previous one with ample evidence tends to influence my decision-making, making it more straightforward.				0.844
6. If strong evidence of a defendant's guilt exists, I consider it a benchmark that, when combined with other aspects of the case, inclines me toward a verdict of guilt.				0.748
11. Relying on the opinions of my colleagues serves as a gold standard for me; the more my viewpoint aligns with theirs, the greater my confidence in my decision.				0.596
Eigenvalues	4.221	1.637	1.218	0.979
Percentage of variance explained by the factors	(35.18%)	(13.65%)	(10.146 %)	(8.16 %)

### 3.2 Descriptive statistics

An exploratory/descriptive data analysis was conducted to get a first impression of the data and assess the normality of distribution in the demographic variables, the four PCs from J-HAQ, and the mean CRT scores. Regarding the use of heuristics, participants seem to report a low frequency of use, whereas performance on CRT was relatively moderate (Table 3).

**Table 3:** Means (*M*), Standard Deviations (*S.D*.), Minimum (Min.) and Maximum (Max.) values of J-HAQ and CRT mean scores.

**Scores**	***M***	***S.D*.**	**Min**.	**Max**.
Availability	7.17	2.86	3	13
Confirmation Bias	5.54	2.48	3	12
Representativeness	5.87	2.13	3	10
Anchoring	11.77	2.44	3	15
CRT	2.94	1.84	0	5

### 3.3 Correlations among factors

Because of the small sample in this study, and because of the deviation from normal distribution in a number of variables, we proceeded with investigating the association between judges' demographic data (educational level, age, and years of experience), their subjective reports of the likelihood of using different types of heuristics (J-HAQ factor scores) and their total performance on the Cognitive Reflection Task, using Spearman's correlation coefficient (ρ). The results revealed a small to medium positive association between education level and reported use of representativeness heuristic (ρ = 0.29, *p* =.04) and a small to medium positive association between CRT mean score and reported use of anchoring heuristic (ρ = 0.29, *p* = 0.04).

Furthermore, there were various statistically significant associations between the reported use of different types of heuristics (Availability—Confirmation Bias: (ρ = 0.43, *p* = 0.002), Availability—Representativeness: (ρ = 0.51, *p* < 0.001), Availability—Anchoring: (ρ = 0.49, *p* < 0.001), Representativeness– Confirmation Bias: (ρ = 0.55, *p* < 0.001).

Based on these correlations, we used G^*^Power (Faul et al., 2009) to also calculate the required sample size for future studies employing the same (or similar) tools, namely the J-HAQ questionnaire and CRT. To achieve sufficient power (1-β = 0.80) and keep error probabilities within the desirable limit (α = 0.05) for one tailed correlations, and given a small to moderate effect size (0.30), a minimum sample of 67 participants is required (Appendix).

### 3.4 Cluster analysis and demographics

**Figure 1:**
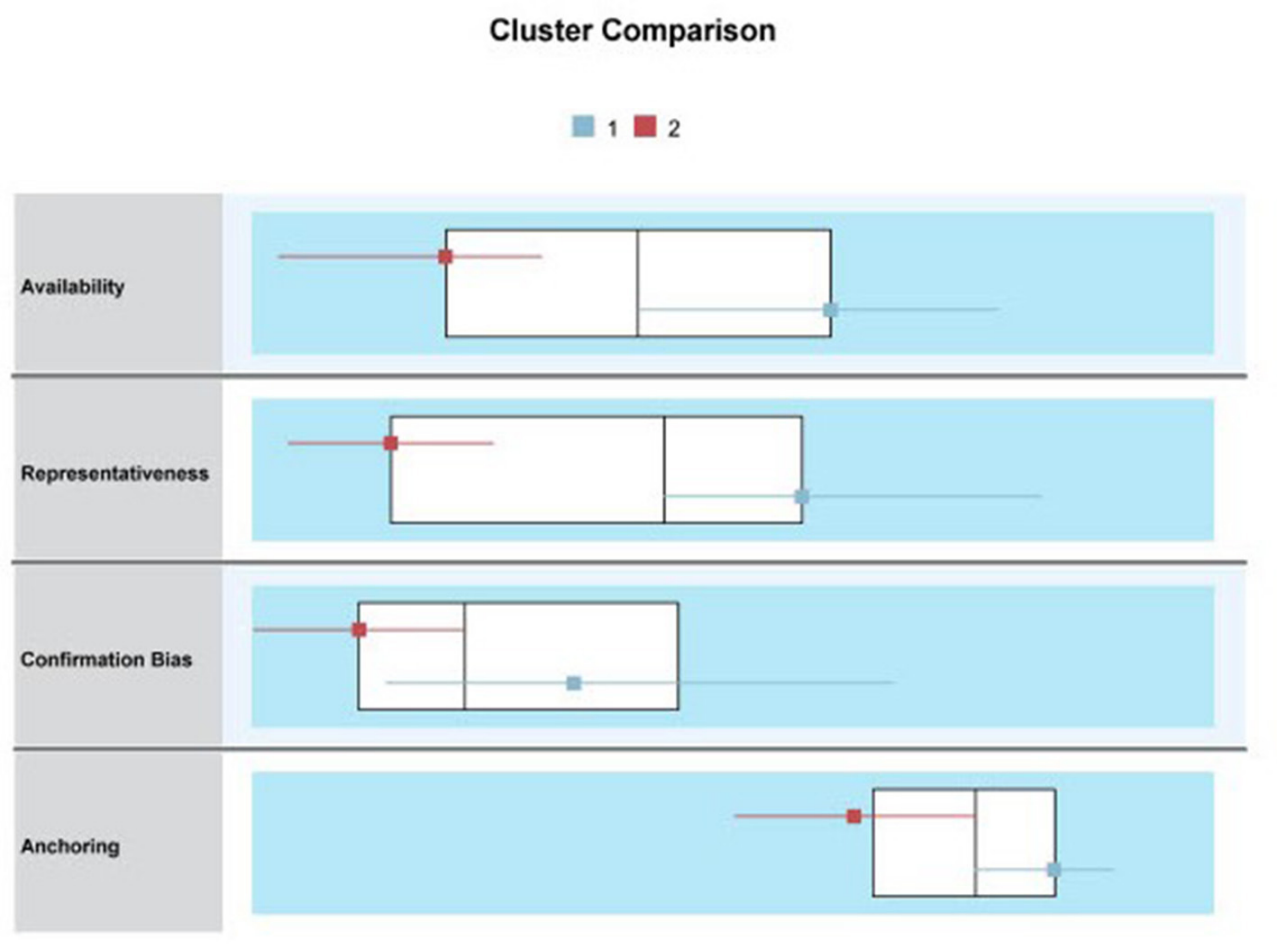
Two clusters of judges with different levels of awareness of the frequency for each type of heuristics use.

Furthermore, expanding on the correlations among some types of heuristics, we conducted a 2-step cluster analysis to examine whether judges' patterns of awareness of using different heuristics gave rise to specific clusters. An analysis using the J-HAQ scores in the reported awareness of using the four types of heuristics revealed two distinct clusters (Figure 1). The first cluster (C1) consisted of 30 participants, and the second (C2) of 22. Both clusters reported a relatively high use of anchoring heuristics (C1: 12.8, C2: 10.36). For the remaining heuristics, C2 with the slightly smaller reported use of anchoring heuristics also reported much less use of availability (4.73) and representativeness heuristics (4.27), as well as lower confirmation bias (4), compared to the equivalent values for C1 (8.97, 7.03, and 6.67 respectively).

When comparing the two clusters for potential differences on demographic variables, using independent samples *t*-tests, we found a significant difference at the educational level (*t*_(40.52)_ = 1.98, *p* = 0.03). Specifically, the cluster with higher awareness of frequency of heuristics use had a slightly higher educational level (M_h_ = 1.77 (SD = 0.43) > M_l_ = 1.5 (SD = 0.51). On the contrary, there were no statistically significant differences in demographic variables such as gender (*t*_(50)_ = −0.26), age (*t*_(50)_ = −0.66), and years of vocational experience (*t*_(50)_ = −0.74) (all *p*s > 0.05), as well as in CRT scores (*t*_(50)_ = 0.87, *p* > 0.05).

## 4 Discussion

The present study pursued three primary objectives. First, we attempted to develop a self-report questionnaire designed to assess the frequency and awareness of judges' utilization of classic cognitive heuristics as described in the existing literature. We aimed to assess its construct validity and internal consistency through rigorous evaluation. Secondly, we delved into examining the correlation between judges' self-reported use of heuristics and their performance on reflective analytical thinking tasks. Lastly, we aimed to assess the associations of demographic variables such as age, gender, educational level, and years of professional experience, with judges' perceived utilization of heuristics and reflective analytical thinking and evaluate naturally occurring patterns of the use of heuristics in the judicial setting.

### 4.1 Assessment of perceived heuristics use in judicial decision-making

Starting with the psychometric characteristics of the J-HAQ, the results of our principal component analysis revealed that items were categorized into four distinct factors representing well-established cognitive heuristics mentioned in relevant literature (Olaborede and Meintjes-Van der Walt, 2020; Peer and Gamliel, 2013): availability, representativeness, anchoring, and confirmation bias. All factors together explained a high percentage of variance. The availability factor emerged as a pure factor based on the intended item categorization. The confirmation bias factor seemed to merge items intended to represent confirmation and overconfidence biases. Additionally, a representativeness heuristic emerged, based on probabilistic judgments, along with the anchoring heuristic. Thus, while the factors extracted from the J-HAQ were equal in number to the theoretically defined ones, their content differed slightly from our initial hypothesized categories.

In any case, the items within each factor were conceptually associated and exhibited high correlations. For example, the lack of a pure overconfidence factor in our study may be attributed to the fact that professions of high prestige require a significant level of confidence in the skills developed through experience and training. This can operate in a complex manner in a high-ranking professional environment, such as that of a judge (Berthet, 2022; Moore and Healy, 2008). Additionally, the representativeness heuristic seems to be relevant to evaluating the probability of specific events by comparison of their characteristics with a particular category. Such a comparison can determine whether the events are typical representatives of this category. In the judicial procedure, ignoring the statistical likelihood in favor of similarity, might lead one to overlook or downplay the base-rate statistics indicating the probability that a crime or event actually occurred (Olaborede and Meintjes-Van der Walt, 2020). Such a bias is dangerous, and it might result in erroneous convictions and sentencing, something that the judges in our study appeared to be aware of.

Additionally, our participants revealed intriguing insights into the establishment of anchoring points, drawing from both specific case evidence and the perceived standards set by their colleagues. This finding resonates with previous research, including a German investigation into the impact of a journalist's phone call as an anchoring influence on judicial decision-making. In this particular study, 23 judges and 19 prosecutors were presented with details of an actual rape case and were subsequently queried by the journalist about the anticipated sentence for the defendant, in comparison to 1/3 year(s). Judges exposed to the low anchor proposed a prison term of 25 months, while those subjected to the high anchor recommended 33 months—a notable increase of 32% (Englich et al., 2006). Furthermore, our study underscores the pivotal role of the anchoring heuristic in judicial proceedings, a finding also corroborated by our cluster analysis results, where both clusters exhibited a significantly higher frequency of anchoring heuristic use compared to other heuristics. Indeed, a substantial body of literature has delved into the anchoring heuristic, showcasing its pervasive influence in relevant contexts (Berthet, 2022; Bystranowski et al., 2021; Olaborede and Meintjes-Van der Walt, 2020). Notable studies, as highlighted by Holier (2017), have demonstrated how conviction demands can serve as potent anchors, shaping final sentencing decisions, often overriding other relevant factors such as the seriousness of the offense or the defendant's criminal history. Moreover, judges' experience did not seem to mitigate the anchoring effect, and sentence decisions were susceptible to influence by exogenous factors.

The reliability coefficients of the J-HAQ were consistent with the typical criteria required in studies utilizing self-reports. Comparable findings were reported in a recent psychometric study by Partsounidou et al. (2023), where the validity and reliability of a self-report measure assessing heuristic usage in medical decision-making were evaluated among a sample of 162 Greek physicians across various specialties. Even though that study had a larger sample size and more items in each factor, the reliability coefficients for both resulting factors (confirmation/overconfidence heuristic, consisting of four items, and anchoring/availability heuristics, comprising five items) were marginally acceptable (α ≈ 0.60).

### 4.2 CRT scores and the correlation of analytical thinking and perceived heuristic use

In order to objectively assess judges' analytical thinking, the Cognitive Reflection Test (CRT) was utilized, as a direct and objective cognitive task requiring participants to overcome initial intuitive judgments to provide correct answers (Toplak et al., 2014). The judges in our sample achieved moderate performance on the CRT, similar to a study by Sinayev and Peters (2015), who administered the CRT to a sample of 1,413 individuals from the general population in the US using the internet. In that study, 57% of the answers were incorrect due to intuitive thinking, 15.3% were attributed to alternative reasoning errors, while only 27.8% were accurate, as a result of analytical thinking (System 2). These scores highlight the challenge of overcoming prepotent intuitive responses in decision-making processes. Even though the CRT's content isn't oriented toward judicial decision-making, it has been employed in previous studies both as a predictor and predicted variable in heuristic decision-making research. In a study by Toplak et al. (2014), it was the only statistically significant predictor of various heuristics and cognitive bias performance tasks, among various measures of cognitive ability, thinking disposition and executive function tasks. On the other hand, Lucena et al. (2021), report its correlation with numerical decision-making, such as insensitivity to base rate. Therefore, a plausible explanation for the positive correlation of CRT scores and self-reported anchoring usage in our study is that this heuristic often relies on an anchor (sometimes numerical) stored in memory, frequently recalled when judging similar events. The CRT's mathematical structure might activate an automatic response from memory, akin to simpler mathematical problems, serving as an anchor that must be overridden to reach a correct solution.

It is worth pointing out that previous research studies reported a negative association between direct assessments of heuristic use and reflective thinking (Toplak et al., 2014; Toplak and Rizeq, 2020; Lucena et al., 2021). Our study differs from those studies, because we did not use objective assessment of heuristics but self-reports. As highlighted in the introduction, these self-reports are deemed reflective of judges' metacognitive knowledge of themselves about the heuristics they employ during judicial decision-making. A small to medium positive correlation was identified between the perceived use of anchoring and CRT performance, partially confirming the second hypothesis. Studies exploring the relationship between metacognitive awareness and decision-making are in their early stages and have garnered recent interest among researchers. There is only one previous study that has examined this relationship to some extent: using self-reports, Colombo et al. (2010) assessed beliefs about decision-making in professional life, investigating metacognitive awareness across different domains of cognitive knowledge, such as tasks, strategies, emotions, and personal attributes. The results of their study indicated that participants were able to perceive and monitor the different mental processes involved in their professional decision-making. Researchers also found that increased metacognitive awareness was particularly linked to situations where automated decision-making processes, such as heuristics, led to errors. However, direct questions about one's knowledge can also be influenced by various factors such as hypothetical reasoning, inference processes, psychological traits, personality characteristics, and motivational aspects. Consequently, estimates derived from such questions might in some cases not always accurately reflect one's actual abilities (Kostaridou-Efklidi, 2011). Taking this into account, we hope that future research will shed light on this relationship and elucidate the exact mechanisms underlying it.

### 4.3 Patterns in judges' reported awareness of heuristics decision-making and associated variables

In our investigation of various factors including gender, age, level of education, and years of experience among judges, we found that only level of education (Bachelor's, Master's, Ph.D.) exhibited a significant and positive correlation with the representativeness heuristic. Our findings suggest that judges with higher educational attainment demonstrate greater awareness and readiness to use the representativeness heuristic, while concurrently acknowledging their susceptibility to the effects of this heuristic (Olaborede and Meintjes-Van der Walt, 2020). Representativeness involves estimating the probability of a particular event based on its similarity to other events rather than its likelihood. In general, higher levels of education are associated with a better understanding of scientific methods, statistical interpretation, and critical thinking (Fruehwald, 2022; Golden, 2023). The results from our cluster analysis further support the significance of educational level in distinguishing between judges who frequently employ heuristics and those who do so less often. Our findings are therefore consistent with Nisbett et al. (1983) results, where expertise was shown to reduce reliance on the representativeness heuristic, especially in examples involving statistical/probabilistic reasoning, as well as with Gigerenzer's (2015) suggestions of LIME with regards to the efficient use of heuristics under the right circumstances. Moreover, recent research findings suggest that individuals with higher cognitive abilities may not rely on System 2 to correct erroneous automatic intuitions. Instead, they tend to demonstrate more accurate intuitive heuristic thinking (Bago and De Neys, 2019). This is further supported by findings indicating that cognitively skilled individuals are more likely to generate correct intuitive responses (Raoelison et al., 2020).

While representativeness heuristic may yield accurate results in judicial decisions, an overreliance on it could also lead to biases, such as when witnesses or defendants are judged based on their demeanor, physical appearance, or racial background, aligning with stereotypes. Metacognitive awareness is therefore a decisive factor for the effective use of such heuristics. Metacognitive thinking involves self-reflection and self-correction, contributing to higher levels of excellence in legal reasoning, a quality often observed in individuals with advanced education. Legal education and the cultivation of metacognitive awareness have been recognized as transformative, enabling judges to observe their thinking processes and identify biases (Lee, 2019). For this reason, significant efforts are underway to introduce interventions aimed at enhancing awareness beginning at the undergraduate level. In general, increased awareness is believed to aid judges in adaptively utilizing heuristics and mitigating cognitive biases during judicial decision-making (Greene and Ellis, 2007; Olaborede and Meintjes-Van der Walt, 2020; Peer and Gamliel, 2013).

### 4.4 Potential applications for dealing with the negative effects of heuristics and other types of noise in the judicial setting

Given the complex nature of the association between noise and bias and/or use of heuristics, further studies are needed. To demonstrate the complex association between the two factors but also their independence, Kahneman et al. (2021) suggest that from noise (inconsistency) we can infer that error is present; but lack of noise does not imply an absence of error. Furthermore, these researchers conceptualize the classification of different types of noise in the judicial system, such as level noise (e.g., differences in the average severity or leniency among judges), pattern noise (such as individual judges responding differently to specific case characteristics), and occasion noise (such as temporal or context dependent variations in judges' decisions) (Danziger et al., 2011; Mustard, 2001).

Interestingly, on their review of this book by Kahneman et al. (2021), Gilhooly and Sleeman (2022) point out that System 1 thinking is prone to occasion noise due to factors like mood and recent experiences, while System 2 is less affected by occasion noise but may introduce system noise if judges apply different rules or standards. Other researchers also point out that System-2 thinking is more likely to demonstrate plan-based judgment errors (namely, a wrong plan leading to error), while System-1 thinking might be more prone to action-based judgment errors (namely, a correct plan wrongly implemented) (Hollnagel, 1993; Reason, 1990). Therefore, noise in the judicial process arises not just from biases, but also from variability in how individuals process information and how they use these two Systems, leading to inconsistent judgments. Further studies that take into account individual differences will promote understanding of these complex issues and allow for more targeted and effective remedial approaches.

The fact that a lot of studies, so far, focus more on biases rather than other sources of noise, might be related to the higher level of difficulty to mitigating the effects of noise, compared to biases. Still, as Kahneman et al. (2021) suggest, some methods with the potential to mitigate noise do exist. Such methods include implementing decision hygiene (breaking down complex judgments into smaller, simpler judgments, use of standardized policies and protocols, minimization of subjectivity, introduction of review mechanisms), utilizing novel technologies such as algorithms and predictive tools, and resorting to training to boost awareness (Curley et al., 2022; Rehavi and Starr, 2014; Spohn and Beichner, 2000). For example, judges might be trained to attend to all relevant aspects of the items to be judged, and make sure that they allocate a similar weight to all critical aspects of a case. Overall, successful reduction of noise is expected to also reduce the average error of judgements.

## 5 Conclusions

To conclude, our study comes as an initial attempt to assess heuristic use among judges with J-HAQ, a self-report instrument measuring their reported frequency of utilization. Unlike objective assessment tasks, the J-HAQ also captures levels of metacognitive awareness in terms of judges' knowledge about their decision-making processes. While higher metacognitive awareness was found to be associated with improved performance on analytical decision-making tasks, this relationship was relatively weak and evident only in the case of anchoring, the most frequently used heuristic among judges. Further research is needed, to understand this intriguing relationship of heuristics use, metacognitive awareness and decision-making. Notably, diverging from research findings on a negative association between cognitive performance on heuristic tasks and analytical thinking, our study that assesses heuristics using self-reports provides evidence for a positive association between the two, potentially reflecting reliance on metacognitive knowledge rather than actual performance. This approach offers invaluable insights into judges' subjective perceptions and awareness of their decision-making strategies, providing data that complements task-based cognitive assessments. Moreover, it sets the ground for evidence-based metacognitive interventions already gaining traction in legal education. Tailoring metacognitive training to the students' needs, as diversified by their varying levels of metacognitive awareness, could facilitate positive change.

A limitation in our study comes from its sample size, and the subsequent caution on external validity of our findings. Given the relatively small size of this specialized population of judges in Greece, recruitment is inevitably challenging. Future research with a larger sample size could mitigate this limitation and increase confidence in our results. A further limitation is the focus on a limited set of heuristics—specifically, availability, confirmation bias, representativeness, and anchoring—potentially overlooking other relevant cognitive biases that may influence judicial decision-making. Future research could address this limitation by incorporating a broader range of cognitive heuristics and biases, such as hindsight bias, framing effects, or the Dunning-Kruger effect, to provide a more comprehensive understanding of heuristic thinking in judicial contexts. Moreover, future studies need assess convergent validity with objective measurements of heuristics, estimate test-retest reliability, and support findings with further confirmatory factor analyses.

Another limitation of this study is its reliance on self-reported data from the J-HAQ, which may introduce self-reporting biases. For instance, in self-reports, participants may respond differently to questions about their behaviors or decision-making based on motivations or a desire to provide socially acceptable or normative answers (Vesely and Klöckner, 2020). Responses may not fully reflect honesty or objectivity but rather align with what is perceived as socially desirable. Additionally, self-reports often show low correlations with actual performance, as participants may describe typical behavior rather than their best efforts (Dang et al., 2020). Therefore, future studies should focus on triangulating these findings with additional methods, such as behavioral experiments or longitudinal studies, to strengthen the validity of the results.

For now, a cautious interpretation of our present findings is advised, until further research corroborates and enhances the robustness of our conclusions. Nevertheless, the development of J-HAQ, with its relatively clear factorial structure, provides researchers with a reliable tool to measure reported heuristics use by judges in future studies. Findings of significant correlation of some of its subscales (here anchoring heuristic) with objective measures of reflective analytical thinking (CRT) and key demographic variables, such as education level, can pave the way for further studies to understand the nature of these associations. Finally, the discovery of clusters with different profiles, and different levels of awareness in using heuristics, highlights the theoretical value of further studies on meta-cognitive processes, as well as the practical value of applying this knowledge in real-world settings such as the judicial domain.

## Figures and Tables

**Figure A1:**
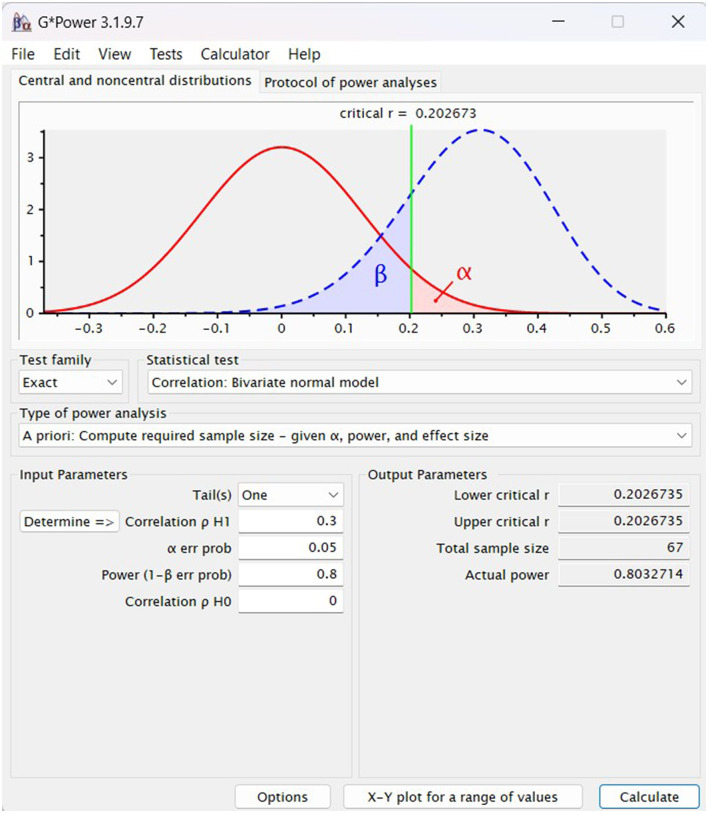
G*Power calculation.

## Data Availability

The raw data supporting the conclusions of this article will be made available by the authors, without undue reservation.
